# E-Cigarette Affects the Metabolome of Primary Normal Human Bronchial Epithelial Cells

**DOI:** 10.1371/journal.pone.0142053

**Published:** 2015-11-04

**Authors:** Argo Aug, Siiri Altraja, Kalle Kilk, Rando Porosk, Ursel Soomets, Alan Altraja

**Affiliations:** 1 Department of Biochemistry, Institute of Biomedicine and Translational Medicine, University of Tartu, The Centre of Excellence for Translational Medicine, Tartu, Estonia; 2 Department of Biomedicine, Institute of Biomedicine and Translational Medicine, University of Tartu, Tartu, Estonia; 3 Department of Pulmonary Medicine, University of Tartu, Tartu, Estonia; 4 Lung Clinic, Tartu University Hospital, Tartu, Estonia; University Hospital Freiburg, GERMANY

## Abstract

E-cigarettes are widely believed to be safer than conventional cigarettes and have been even suggested as aids for smoking cessation. However, while reasonable with some regards, this judgment is not yet supported by adequate biomedical research data. Since bronchial epithelial cells are the immediate target of inhaled toxicants, we hypothesized that exposure to e-cigarettes may affect the metabolome of human bronchial epithelial cells (HBEC) and that the changes are, at least in part, induced by oxidant-driven mechanisms. Therefore, we evaluated the effect of e-cigarette liquid (ECL) on the metabolome of HBEC and examined the potency of antioxidants to protect the cells. We assessed the changes of the intracellular metabolome upon treatment with ECL in comparison of the effect of cigarette smoke condensate (CSC) with mass spectrometry and principal component analysis on air-liquid interface model of normal HBEC. Thereafter, we evaluated the capability of the novel antioxidant tetrapeptide O-methyl-l-tyrosinyl-γ-l-glutamyl-l-cysteinylglycine (UPF1) to attenuate the effect of ECL. ECL caused a significant shift in the metabolome that gradually gained its maximum by the 5^th^ hour and receded by the 7^th^ hour. A second alteration followed at the 13^th^ hour. Treatment with CSC caused a significant initial shift already by the 1^st^ hour. ECL, but not CSC, significantly increased the concentrations of arginine, histidine, and xanthine. ECL, in parallel with CSC, increased the content of adenosine diphosphate and decreased that of three lipid species from the phosphatidylcholine family. UPF1 partially counteracted the ECL-induced deviations, UPF1’s maximum effect occurred at the 5^th^ hour. The data support our hypothesis that ECL profoundly alters the metabolome of HBEC in a manner, which is comparable and partially overlapping with the effect of CSC. Hence, our results do not support the concept of harmlessness of e-cigarettes.

## Introduction

Electronic cigarettes or e-cigarettes as the major representative of the electronic nicotine delivery systems are rapidly gaining popularity, because they are trivially expected to be a relatively harmless alternative for conventional smoking [1]. In spite of being marketed as “cigarettes”, technically, e-cigarettes are rather similar to multi-dose inhalers, nebulizers, or other equipment designed for inhalable drug delivery [2]. The typical composition of the e-cigarette filling liquid includes mainly propylene glycol, vegetable glycerine, nicotine, and flavorings [3]. E-cigarettes have already been proposed as a promising tool for smoking cessation [4–6], but, on the other hand, they are deemed as an attractive gateway into tobacco consumption and renormalization of smoking among the youth [7, 8]. It is likely that the usage of e-cigarettes is associated with an exposure to a somewhat smaller number of toxicants compared to that resulting from consumption of combustible tobacco products [9]. However, considering the relatively recent entry of e-cigarettes into the market and the delay for onset of many diseases, such as chronic obstructive pulmonary disease (COPD) or lung cancer, conclusive evidence about the possible connection between e-cigarettes and such diseases will not be available for many forthcoming years [10]. Although in short-term studies, e-cigarettes have been shown to harm lung function to a lesser extent than does conventional smoking at a similar level of nicotine bioavailability [11], they are not harmless. An increase has been demonstrated in peripheral airway resistance after using e-cigarette and proposed escalating oxidative stress as one of the deleterious effects of the use of e-cigarettes [12], analogously to that rising from the combustive cigarettes.

Metabolomics represents a rapidly emerging technology to assess the instant effects of external stimuli on biological systems, while monitoring the changes in the levels of metabolites, but so far, usage of this technique has been unobtrusive in assessing the safety of e-cigarettes. We have recently described the metabolic changes of primary normal human bronchial epithelial cells, differentiated in air/liquid interface, induced by cigarette smoke condensate and the extent in which these changes are modified by antioxidants [13]. Since epithelial cells are the immediate target of inhaled toxicants, we hypothesized that e-cigarette liquid may affect the metabolome of HBEC in a manner comparable of the influence of the smoke from ordinary cigarettes and that the changes, once detected, are at least in part induced by oxidant-driven mechanisms. To test these hypotheses, we designed an untargeted metabolomics study to unravel the metabolic changes induced by e-cigarette liquid (ECL) on primary normal human bronchial epithelial cells (HBEC) and to evaluate the capability of an antioxidant glutathione analogue UPF1 (O-methyl-l-tyrosinyl-γ-l-glutamyl-l-cysteinylglycine) to ameliorate the changes.

## Methods

### Reagents

“Strong/high” AIRSmoke ECL was from AIR SMOKE LLC (Riga, Latvia). Cigarette smoke condensate (CSC), prepared from the University of Kentucky 3R4F standard research cigarettes on an FTC-adapted Smoke Machine from Murty Pharmaceuticals (Lexington, KY), was dissolved in dimethyl sulfoxide at 40 mg/ml. UPF1 was synthesized as described before [14]. N-acetylcysteine (NAC) and solvents for mass-spectrometry (methanol, water, and formic acid) were from Sigma-Aldrich (St Louis, MO).

### Cell Culture and Treatments

Primary normal HBEC were purchased from Lonza (Lonza Ltd., Basel, Switzerland) and cultured in an air-liquid interface (ALI) system, as described previously [13], to undergo mucociliary cell differentiation. Briefly, after expansion, HBEC (passage 3) were seeded onto cell culture inserts (0.4 μm pore size, BD Biosciences, Bedford, MA), coated with type I rat tail collagen (BD Biosciences) and grown submerged in B-ALI™ growth medium (Lonza). Once a fully confluent monolayer was established, the cell cultures were shifted to ALI conditions by removing the culture medium from the apical compartment and replacing with B-ALI™ differentiation medium supplemented with inducer (Lonza) in the basal chamber. Thereafter, the cells were cultured at ALI for 16 days under a humidified 5% CO_2_ atmosphere at 37°C with refreshing the medium in the basolateral compartment every 48 h before the cells were used in experiments.

The cells were exposed either to ECL diluted to 100 μM by nicotine or to 10 μg/ml CSC for 1, 5, 7, and 13 h. The test compounds were added to the basolateral media (500 μl), as well as onto the apical surface (25 μl). To test the effect of the antioxidants, 10 μM UPF1 or 2 mM NAC were added to the cells pre-exposed for 1 h to the ECL (100 μM by nicotine) or CSC (10 μg/ml) and incubated additionally for 1, 4, 6, and 12 h in the presence of the initially added ECL or CSC. After incubation, the cells were washed with PBS and lysed with 3 freeze-thaw cycles in triple-distilled water.

### Mass Spectrometry

Proteins were precipitated from the cell lysates with 75% methanol and centrifuged for 15 min at 15,800 × g. The supernatants were analyzed on Q-Trap 3200 mass spectrometer (AB Sciex, Framingham, MA) to obtain the full spectra in positive and negative enhanced mass scan mode. Samples (50 μl) were injected and analyzed in isocratic flow of 0.05 ml/min water, 0.15 ml/min methanol, and 0.1% formic acid. The ionspray voltage, declustering potential and entrance potential were 4,500 V, 20 V, and 10 V, respectively. In negative scans, the respective negative voltages were applied. Mass-to-charge ratios (m/z) from 90 to 1,000 were detected which is the mass range of most likely identifiable metabolites. Collision energy from 10 to 60 V was used in fragmentation analyses, since optimal characteristic fragment spectra are obtained in that range.

### Data Analysis

The signals of samples were analyzed as fold changes from the respective signals in the untreated cells. The fold changes were calculated as (sample intensity/control intensity)-1 for increase and 1-(control intensity/sample intensity) for decrease, to have the increased and decreased signals on the same scale. Principal component analysis (PCA) was used to detect and to robustly illustrate the general treatment-related time-dependent variances, based on the full mass spectra of all samples. T-test was conducted on logarithmically transformed data to estimate how large fraction of the whole detectable metabolome was affected by ECL, CSC, UPF1, and NAC. Pearson’s correlation coefficients between all signals at different time points and under different treatments were calculated. Signals of the spectra were congregated by their pattern of dynamics under the influence of ECL and/or CSC focusing on the 1–7 h time points to capture the most proximate effects of both the ECL and CSC. For identification of the metabolites, fragmentation analysis was conducted for the signals, which were significantly (p<0.05) altered by ECL and/or CSC or had a correlation coefficient above 0.9 to the signals significantly altered by ECL and/or CSC. The fragmentation spectra were compared to our in-house library and public metabolome databases (http://www.hmbd.ca, http://metlin.scripps.edu, http://www.massbank.jp/ and http://www.lipidmaps.org). All statistical analyses were performed with R version 2.14.0 (The R Foundation for Statistical Computing, Vienna, Austria).

## Results

### Effect of ECL and CSC on Metabolome of HBEC

**Fig 1:**
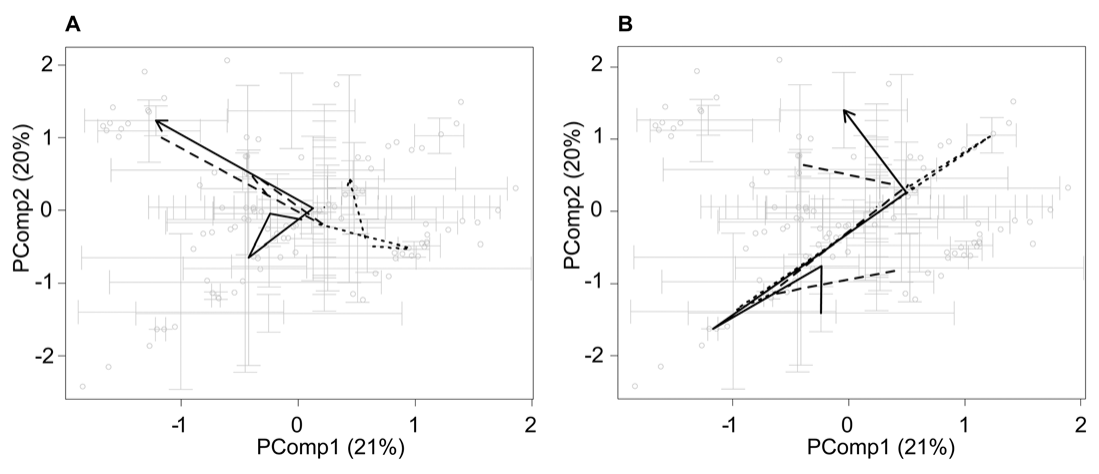
Principal component analysis of the metabolic state of primary normal human bronchial epithelial cells cultivated in air-liquid interface after exposure to (A) e-cigarette liquid (ECL) (100 μM by nicotine) and (B) 10 μg/mL cigarette smoke condensate (CSC) (black solid lines). The time points at the arrow turns are 1, 2, 5, 7, and 13 h. Long dashes: Addition of 10 μM O-methyl-l-tyrosinyl-γ-l-glutamyl-l-cysteinylglycine to the cells having been exposed to ECL for 1 h (the arrow starts at 2 h time point), short dashes: Addition of 2 mM N-acetylcysteine to the cells having been exposed to ECL for 1 h (the arrow starts at 2 h time point). For each treatment and for any time point, n = 3 (gray circles). Error bars indicate standard errors of means. PComp1 and PComp2 are the two principal components describing the highest fraction of the effect of cells’ metabolome.

PCA revealed that ECL caused fluctuations that gradually gained their first maximum by the 5^th^ h, which has then receded by the 7^th^ h (Fig 1A). The second deviation in a different direction occurred in the ECL-stimulated cells at 13^th^ h (Fig 1A). Fluctuations in the metabolome caused by CSC were in the scale and directions comparable with those caused by the ECL (Fig 1B). A remarkable initial deviation was gained already by the 1^st^ h after CSC treatment. The absolute maximal deviation of the metabolome was achieved after 5 h from the start of the CSC treatment and was more extensive than that caused by ECL. The metabolic state of the cells stimulated by CSC returned to that of untreated cells by the 7^th^ h. By the 13^th^ h, however, the second deviation in another direction, analogous to that observed following the ECL-stimulation, was reached.

**Fig 2:**
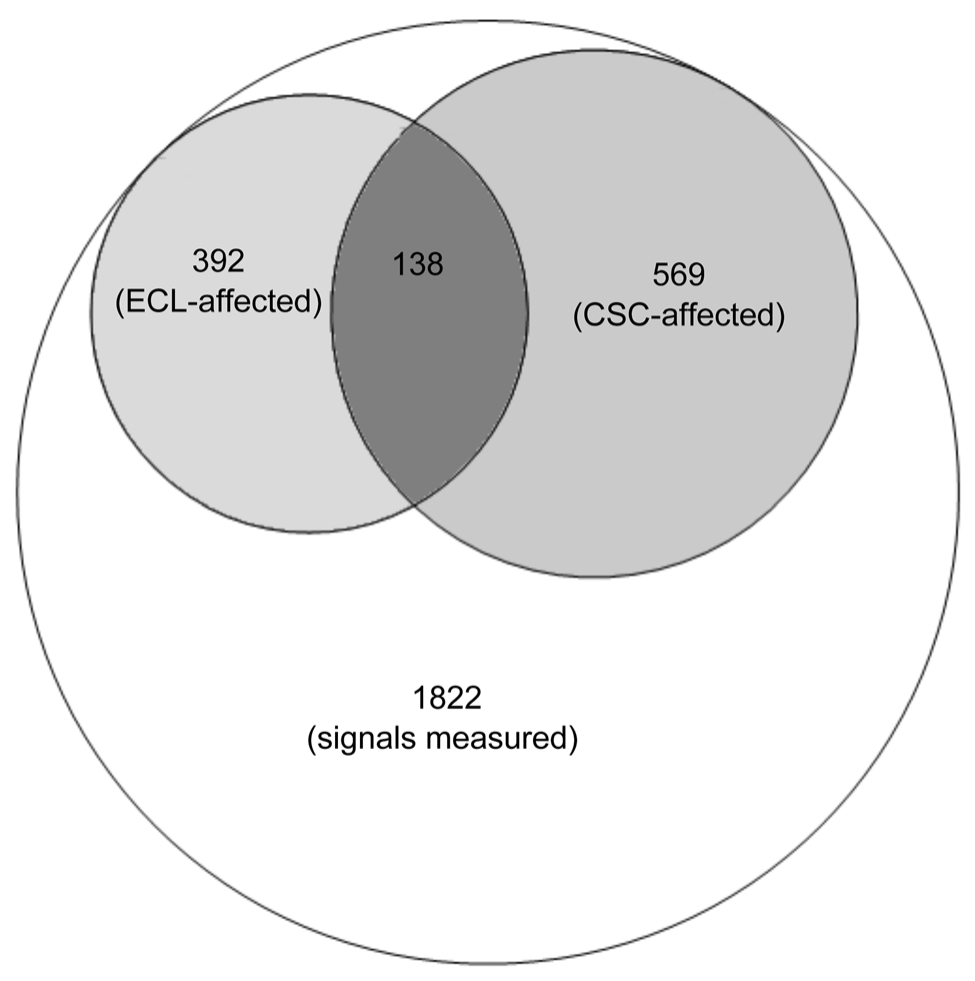
Mass spectrum of primary normal human bronchial epithelial cells cultivated in air-liquid interface after exposure to e-cigarette liquid (ECL) (100 μM by nicotine) and 10 μg/mL cigarette smoke condensate (CSC) consisting of 1,822 distinct mass-to-charge signals. The figure represents the proportions of the spectrum that were significantly (p*<*0.05) affected by addition of ECL (392 signals) or CSC (569 signals) during the first 7 h. There were 138 signals that were significantly affected by both stimuli.

Each mass spectrum consisted of 1,822 signals. Out of these, ECL and CSC alone were found to significantly alter 392 and 569 signals, respectively, while 138 signals were altered by both ECL and CSC at least at one time point during the first 7 hours (Fig 2). The highest number of signals that were significantly increased or decreased in the presence of both ECL and CSC was found at 1 h after addition of ECL or CSC onto the cells (Table 1). Conversely, the lowest number of altered signals was found at 7 h and at 13 h in case of stimulation with ECL and CSC, respectively (Table 1).

**Table 1:** The numbers of mass spectrometry signals (mass-to-charge ratios) from the lysate of primary normal human bronchial epithelial cells cultured in air-liquid interface significantly (p*<*0.05) altered by e-cigarette liquid (ECL) (100 μM by nicotine) or 10 μg/mL cigarette smoke condensate (CSC) at the respective time points.

	Time points					
Number of signals	1 h	2 h	5 h	7 h	13 h	
Signals altered by ECL only						
Increased	56	48 (2/3)	62 (5/6)	31 (8/7)		66 (5/2)
Decreased	101	58 (7/5)	59 (11/6)	20 (7/2)		80 (3/10)
Signals altered by both ECL and CSC						
Increased by both ECL and CSC	7	7	10	2		3
Reduction of the effect of both stimuli		(0/0)	(0/0)	(0/0)		(0/0)
Reduction of the effect of ECL only		(0/0)	(0/0)	(2/1)		(0/0)
Reduction of the effect of CSC only		(0/0)	(1/0)	(0/0)		(1/1)
Decreased by both ECL and CSC	14	3	6	1		2
Reduction of the effect of both stimuli		(0/0)	(0/0)	(0/0)		(0/0)
Reduction of the effect of ECL only		(0/1)	(0/0)	(0/0)		(1/0)
Reduction of the effect of CSC only		(0/0)	(0/0)	(0/0)		(0/1)
Increased by ECL and decreased by CSC	0	0	0	0		4
Reduction of the effect of both stimuli		(0/0)	(0/0)	(0/0)		(0/0)
Reduction of the effect of ECL only		(0/0)	(0/0)	(0/0)		(0/0)
Reduction of the effect of CSC only		(0/0)	(0/0)	(0/0)		(0/1)
Decreased by ECL and increased by CSC	5	1	1	0		2
Reduction of the effect of both stimuli		(0/0)	(0/0)	(0/0)		(0/0)
Reduction of the effect of ECL only		(0/0)	(0/0)	(0/0)		(0/0)
Reduction of the effect of CSC only		(0/0)	(0/0)	(0/0)		(0/0)
Signals altered by CSC only						
Increased	84	50 (0/2)	85 (11/1)	86 (15/5)		61 (4/9)
Decreased	160	57 (6/4)	81 (5/4)	66 (10/5)		36 (11/12)

The numbers of signals in case of which 10 μM O-methyl-l-tyrosinyl-γ-l-glutamyl-l-cysteinylglycine (UPF1)/2 mM N-acetylcysteine (NAC) significantly reduced the effect of the respective stimuli are shown in parentheses.

### Effect of the Antioxidants on the ECL- and CSC-induced Changes

UPF1 exerted its maximal ameliorating effect on the deviation induced by ECL at the 5 h time point (i.e. at 4 h after addition of UPF1 to the cells), which co-occurred with the first maximum of the effect of ECL (Fig 1A). Afterwards, UPF1 did not affect the effect of ECL. In case of CSC, UPF1 counteracted the effect of CSC at the 2 h time point and at the 13 h time point and did not alter the effect of CSC meanwhile (Fig 1B).

In the ECL-treated cells, throughout the experiment, NAC caused a metabolic shift, which was different from that produced by the ECL alone (Fig 1A). In contrast, the fluctuations caused by CSC, however, were not altered by NAC before the 7 h time point (Fig 1B). The reduction of the ECL-induced alterations was maximally induced by both UPF1 and NAC at 7 h, when UPF1 reduced 15 and NAC reduced 9 out of the 51 signals altered by ECL at that time point (Table 1, rows 1–2).

### Identified Signals Altered by ECL and/or CSC

**Fig 3:**
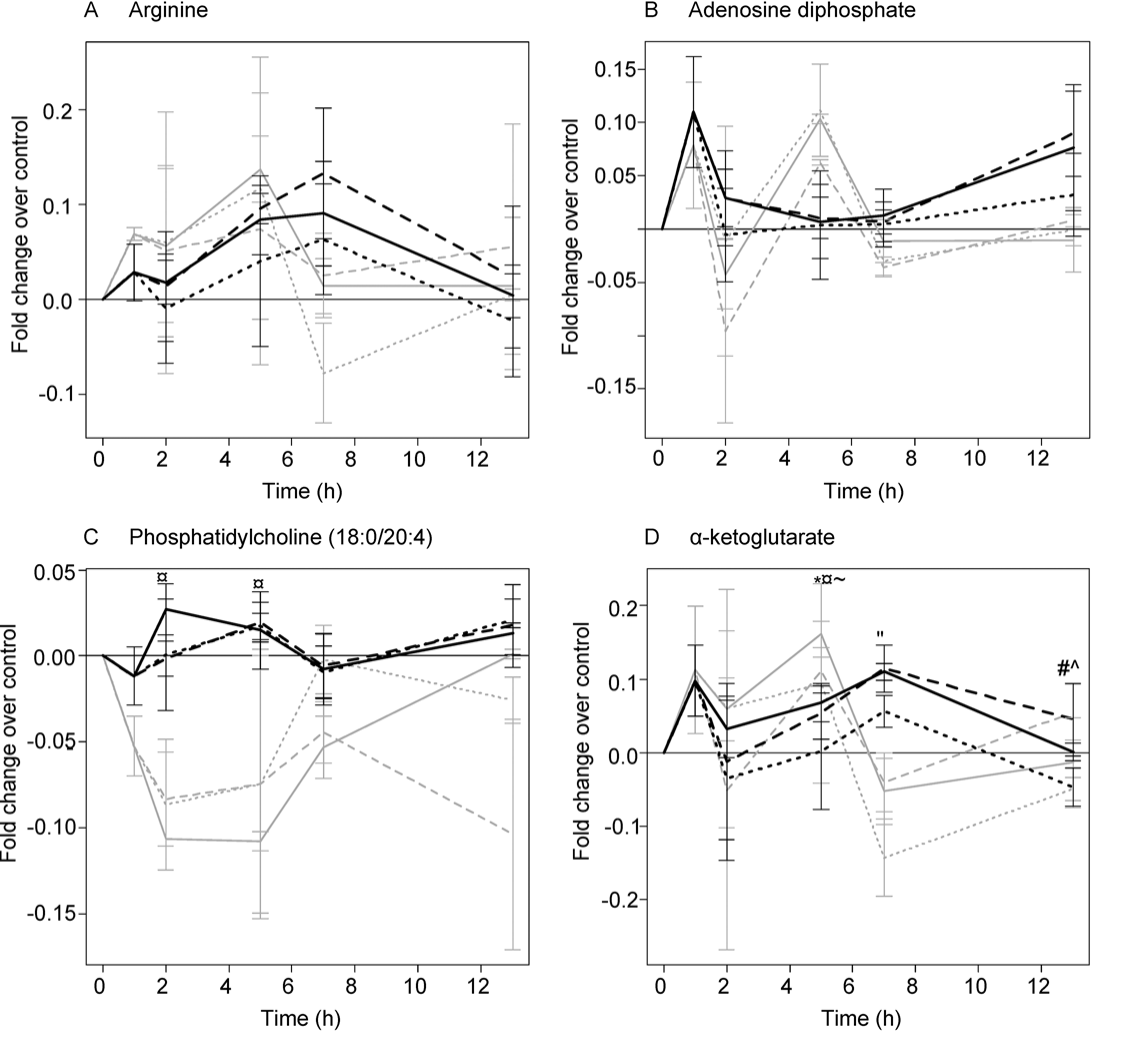
Time dynamics of the expression of the metabolites of primary normal human bronchial epithelial cells (HBEC) cultured in air-liquid interface being affected by e-cigarette liquid (ECL) (100 μM by nicotine) (black lines) and 10 μg/mL cigarette smoke condensate (CSC) (grey lines) for 13 h. Solid lines: HBEC treated with ECL (solid black lines) or with CSC (solid grey lines). Long black dashes: HBEC treated with ECL and 10 μM O-methyl-l-tyrosinyl-γ-l-glutamyl-l-cysteinylglycine (UPF1) (added at 1h). Long grey dashes: HBEC treated with CSC and 10 μM UPF1 (added at 1h). Short black dashes: HBEC treated with ECL and 2 mM N-acetylcysteine (NAC) (added at 1h). Short grey dashes: HBEC treated with CSC and 2 mM NAC (added at 1h). For each treatment and for any time point, n = 3. Error bars indicate standard errors of means. (A) arginine ([M+H]^+^ = 175); (B) adenosine diphosphate ([M-H]^-^ = 426); (C) phosphatidylcholine (18:0/20:4) ([M+H]^+^ = 811); (D) α-ketoglutarate ([M-H]^-^ = 145). *p < 0.05, ECL-exposed cells versus untreated cells; #p < 0.05, ECL- and UPF1-exposed cells versus untreated cells; ^p < 0.05, ECL- and NAC-exposed cells versus untreated cells; ¤p < 0.05, CSC-exposed cells versus untreated cells; ~p < 0.05, CSC- and UPF1-exposed cells versus untreated cells; “p < 0.05, CSC- and NAC-exposed cells versus untreated cells.

ECL exclusively altered statistically significantly the concentrations of arginine (at 5 h, 10% increase) (Fig 3A), histidine (at 1 h, 4% increase) and xanthine (at 1 h, 4% increase) (S1A and S1B Fig). Nicotine was identified as one of the signals in the ECL-exposed cells, while in the CSC-stimulated cells, the increase of nicotine remained under the level of significance throughout the experiment (S1C Fig). Moreover, the nicotine content increased and reached its maximum more rapidly in the ECL-exposed cells, as compared with that occurring as a result of the CSC exposure.

Adenosine diphosphate (at 1 h, 10% increase by ECL; at 5 h, 8% increase by CSC) (Fig 3B), as well as three lipid species from the phosphatidylcholine (PC) family [PC(p-18:0/18:1) (at 1 h, 3% decrease by ECL; at 7 h, 7% decrease by CSC), PC(36:2) (at 2 h, 6% decrease by ECL; at 2 h, 5 h, and 7 h, 7% decrease by CSC), and PC(36:6) (at 1 h and 2 h, 5% decrease by ECL; at 2 h, 4% decrease by CSC)] (S1D–S1F Fig), were identified as metabolites affected significantly by both ECL and CSC.

CSC, but not ECL, altered significantly the levels of PC(18:0/ 20:4) (at 2 and 5 h, 11% decrease), α-ketoglutarate (at 5 h, 14% increase) (Fig 3C and 3D), glutamine (at 5 h, 13% increase), PC(o-16:0/20:4) (at 5 h, 12% decrease), inorganic phosphate (at 7 h, 3% decrease), spermidine (at 13 h, 4% decrease), phosphatidylethanolamine (PE)(38:7) (at 5 h and at 7 h, 6% decrease), creatine (5 h, 8% increase), hypoxanthine (at 2 h and at 5 h, 7% increase), and aconitic acid (at 5 h, 10% increase) (S1G–S1N Fig). Additionally, the level of proline (S1O Fig) correlated with that of histidine (S1A Fig) (*R* = 0.91, p*<*0.001), as did the level of glutamate with that of glutamine (*R* = 0.8; p<0.001).

Out of the identified metabolites that were affected by ECL, neither UPF1 nor NAC was able to significantly modulate the changes induced by ECL (Figs 3 and S1). UPF1 avoided the increase of the level of ADP in the HBEC exposed to CSC, while NAC did not (Fig 3B).

## Discussion

In this study, we unraveled the effect of ECL on the intracellular metabolite composition in HBEC and clarified whether antioxidants could assist the cells in recovering the injury. PCA indicated that the fluctuations caused by ECL were biphasic: the first maximum occurred by the 5^th^ h and the next one by the 13^th^ h (Fig 1A) with intermediate abating the initial fluctuation to reach the status of unexposed cells by the 7^th^ h. This dynamics of the metabolome is in accordance with the number of significantly the ECL-altered signals, which was the lowest at the 7^th^ hour (Table 1, rows 1 and 2). Notwithstanding, at the 1^st^ hour of exposure to ECL, the relatively low fluctuation revealed by PCA coincided with the highest number of significantly altered signals, may imply a controversy. However, since PCA considers all signals of the spectrum regardless of their statistical significance, one could suggest that the amount of metabolites undergoing non-significant changes increases at a slower rate, compared to that of the significantly altered metabolites until gaining equilibrium at the 5^th^ hour. For identification, the signals were currently chosen based on the statistical significance of their alterations. Therefore, the identified metabolites may obviously reflect only a tip of an iceberg in terms of the effect of ECL on HBEC.

The levels of glutamate, glutamine, proline, histidine, and arginine were increased by ECL by the 1^st^ hour. At the 2^nd^ and 5^th^ hour, the contents of these amino acids, although heightened in comparison with untreated cells, were higher in CSC- than in ECL-treated cells, but the magnitude of these differences was low. On the A549 cell line, it has been shown that 4 h exposure of cigarette smoke extract increases the level of aspartic acid, while the gas phase cigarette smoke increases the level of glutamic acid, isoleucine, leucine, asparagine, proline, glutamine, arginine, tyrosine, phenylalanine, histidine, threonine, valine, tryptophan, and methionine [15]. In our current study, the increase of all identified amino acids (arginine, histidine, glutamine, and proline) in the CSC-treated HBEC occurred throughout the first 5 hours. This indicates an alarming similarity with the effect of ECL, which increased the contents of these amino acids as well, although for a shorter period of time. One could argue for that such a shift may reflect an enhanced protein breakdown or intensified protein turnover. A recent study has demonstrated that not only protein damage, but also a reduction of nascent protein synthesis is induced by cigarette smoke [16], which fact, in the light of our current results on the ECL-induced shifts, emphasizes the potential harm caused by e-cigarette use. According to the best of our knowledge, there is no former data in the literature regarding ECL with this regard.

The level of ADP was initially increased by ECL and CSC in the current study. Formerly, an analogous increase has been shown on the A549 cell line with gaseous cigarette smoke [17]. Increased energy demand is an expected response for all cells presented to stress, whereas the reduced ATP/ADP ratio could be a trigger for adaptive reactions, including primarily increased generation of energy. Apart from a non-significant increase in the glucose level (S1Q Fig), the significant changes in the content of creatine, phospholipids, amino acids, and citrate cycle intermediates refer to a linkage to the changed energy management in HBEC in the present study. Activated β-oxidation of fatty acids, as shown to be caused by cigarette smoke [17], is likely to be behind the decrease of phospholipids in our study. Although the decrease of the expression of phospholipids remained shorter with ECL-stimulation than following the CSC-exposure, the initiation of similar changes by ECL at as early as at the 1^st^ h of exposure further highlights the detrimental potential of ECL. The decrease in phospholipids could also be due to augmentation of the arachidonic acid derived-inflammatory pathways. CSC, but not ECL, caused a deep fall of arachidonic acid-containing phosphatidylcholines during 2–7 h. For lipid species that yielded no fragments characteristic of arachidonic acid, the difference was less apparent. Hence, the ECL may be a weaker inducer of phospholipase A_2_ activity than CSC.

Xanthine and hypoxanthine have been shown to decrease in content in the A549 cell line under the influence of cigarette smoke [17]. With CSC-stimulation, we currently detected an increase of both xanthine and hypoxanthine in HBEC. However, hypoxanthine content was not increased by ECL, but was rather decreased during the first 5 hours.

In addition to propylene glycol and glycerol, it has been suggested that different flavorings may account for the impact of e-cigarette. Compounds that are considered safe e.g. in food may not be safe, when inhaled even in similar concentrations during smoking of e-cigarette [18]. Although the content of nicotine in ECL was higher than in CSC used in the experiments due to the nature of the respective compounds, as the CSC is an extraction of the solid phase of the cigarette smoke with nicotine remaining mostly in the gaseous phase, the uptake of nicotine by HBEC was faster with ECL than with CSC (the peak level of nicotine was reached within 1 h with ECL, while with CSC, it took 2 h). Whether inhibition of the natural resistance of the membranes of HBEC by the constituents of ECL resulting in heightened vulnerability to any harming substances/particles that are inhaled during usage of e-cigarette is behind this evidence or whether the rapidity of the increase in the cellular nicotine levels contributes to a quicker development of nicotine addiction in e-cigarette consumers remains to be elucidated in further studies.

Metabolic comparison of the effects of ECL and CSC revealed that although the proportions of the metabolome of HBEC affected by CSC or ECL were similar, the profiles of the changes were not completely alike. During the first hour, ECL and CSC affected, both in a unique way, 24% and 35% of the metabolome, respectively. With both stimuli, a return to the normal metabolism occurred by roughly 7 h, followed by a second alteration at the 13^th^ h. Compared with the initial changes, the second reaction drove the metabolome to a different direction and, interestingly, this was similar between ECL and CSC.

PCA revealed that NAC was more capable of suppressing the metabolic fluctuations caused by ECL or CSC than UPF1 (Fig 1A). Moreover, it seems that the changes in the HBEC metabolome that appeared at 13 h were not that well reduced by the antioxidants, as were those occurring earlier (Table 1). This may indicate that the ECL-induced metabolic changes of “late onset” involve less oxidative stress in relation to other possible metabolic pathways including those linked to energy production/consumption. This is supported by the current dynamics of ADP that tended to increase in content by the 13^th^ h (Fig 3B). On the other hand, comparison between the two antioxidants for their effects on the individual significantly affected signals (Table 1), however, suggested that both antioxidants are roughly equal in terms of their potencies at the particular concentrations used.

It has been noted that the reduced glutathione (GSH) is depleted rapidly by CSC in the A549 [17] and BEAS-2B [13, 19] cell lines following an increase in GSH level as a counter-reaction [20]. This tendency is not transferable to primary normal HBEC, as indicated in the current study (S1P Fig), as well as in our previous work [13]. In the context of HBEC, the dynamics of GSH by CSC is generally consistent with that of ECL undergoing an initial increase and normalizing by the 13^th^ hour (S1P Fig). Both antioxidants tended to normalize the level of GSH.

Inclusion of an acute setting only may serve as a limitation of the study. However, there were changes detected in the HBEC metabolome of similar profoundness in comparison with those caused by the acute exposure to CSC. In the light of the current results and the doubtless deleteriousness of chronic contact to cigarette smoke, long-term use of e-cigarettes may probably hurt the airways rather than remain unhazardous.

In conclusion, our comparative metabolomic study designed to reveal the acute effect of ECL suggests that e-cigarettes have immediate and profound adverse effects on the metabolomic status of the HBEC comparable to those seen with CSC. However, to answer, whether the use of e-cigarettes for any purpose should be avoided, further data from studies addressing the chronicity of the changes caused or even clinical data are needed to obtain the arguments. Although there are similarities between the influences of ECL and CSC, the biological effects of e-cigarettes on the HBEC include aspects, which cannot be directly deduced from the effects of conventional cigarettes. The evidence that antioxidants can alleviate some of the acute ECL-induced changes suggests an involvement of oxidative stress as one of the mechanisms behind the action of ECL.

## Supporting Information

S1 Fig Time dynamics of the expression of the metabolites of primary normal human bronchial epithelial cells (HBEC) cultured in air-liquid interface being affected by e-cigarette liquid (ECL) (100 μM by nicotine) (black lines) and 10 μg/mL cigarette smoke condensate (CSC) (grey lines) for 13 h. Solid lines: HBEC treated with ECL (solid black lines) or with CSC (solid grey lines). Long black dashes: HBEC treated with ECL and 10 μM O-methoxy-Ltyrosinyl-γ-L-glutamyl-L-cysteinylglycine (UPF1) (added at 1h). Long grey dashes: HBEC treated with CSC and 10 μM UPF1 (added at 1h). Short black dashes: HBEC treated with ECL and 2 mM N-acetylcysteine (NAC) (added at 1h). Short grey dashes: HBEC treated with CSC and 2 mM NAC (added at 1h). For each treatment and for any time point, n = 3. Error bars indicate standard errors of means. (A) Histidine ([M+H]+ = 156); (B) xanthine ([M+H]+ = 153); (C) nicotine ([M+H]+ = 163); (D) phosphatidylcholine (p-18:0/18:1) ([M+H]+ = 772); (E) phosphatidylcholine (36:2) ([M+H]+ = 786); (F) phosphatidylcholine (36:6) ([M+H]+ = 778); (G) glutamine ([M+H]+ = 147); (H) phosphatidylcholine (o-16:0/20:4) ([M+H]+ = 768); (I) inorganic phosphate ([M+H]- = 97); (J) spermidine ([M+H]+ = 146); (K) phosphatidylethanolamine (38:7) ([M +H]+ = 762); (L) creatine ([M+H]+ = 132); (M) hypoxanthine ([M+H]+ = 137); (N) aconitic acid ([M-H]- = 173); (O) proline ([M+H]+ = 116); (P) glutathione ([M-H]- = 306; (Q) glucose ([M-H]- = 179). *p < 0.05, ECL-exposed cells versus untreated cells; #p < 0.05, ECL- and UPF1-exposed cells versus untreated cells; ^p < 0.05, ECL- and NAC-exposed cells versus untreated cells; ¤p < 0.05, CSC-exposed cells versus untreated cells; ~p < 0.05, CSC- and UPF1-exposed cells versus untreated cells; "p < 0.05, C- and NAC-exposed cells versus untreated cells.
(PDF)
