# Supplementary material for: E-Cigarette Affects the Metabolome of Primary Normal Human Bronchial Epithelial Cells
Source: PLoS One. 2015 Nov 4;10(11):e0142053. doi: 10.1371/journal.pone.0142053 (PMC4633226; doi:10.1371/journal.pone.0142053)
Supplement: S1 Fig — Solid lines: HBEC treated with ECL (solid black lines) or with CSC (solid grey lines). Long black dashes: HBEC treated with ECL and 10 μM O-methoxy-Ltyrosinyl-γ-L-glutamyl-L-cysteinylglycine (UPF1) (added at 1h). Long grey dashes: HBEC treated with CSC and 10 μM UPF1 (added at 1h). Short black dashes: HBEC treated with ECL and 2 mM N-acetylcysteine (NAC) (added at 1h). Short grey dashes: HBEC treated with CSC and 2 mM NAC (added at 1h). For each treatment and for any time point, n = 3. Error bars indicate standard errors of means. (A) Histidine ([M+H]+ = 156); (B) xanthine ([M+H]+ = 153); (C) nicotine ([M+H]+ = 163); (D) phosphatidylcholine (p-18:0/18:1) ([M+H]+ = 772); (E) phosphatidylcholine (36:2) ([M+H]+ = 786); (F) phosphatidylcholine (36:6) ([M+H]+ = 778); (G) glutamine ([M+H]+ = 147); (H) phosphatidylcholine (o-16:0/20:4) ([M+H]+ = 768); (I) inorganic phosphate ([M+H]- = 97); (J) spermidine ([M+H]+ = 146); (K) phosphatidylethanolamine (38:7) ([M +H]+ = 762); (L) creatine ([M+H]+ = 132); (M) hypoxanthine ([M+H]+ = 137); (N) aconitic acid ([M-H]- = 173); (O) proline ([M+H]+ = 116); (P) glutathione ([M-H]- = 306; (Q) glucose ([M-H]- = 179). *p < 0.05, ECL-exposed cells versus untreated cells; #p < 0.05, ECL- and UPF1-exposed cells versus untreated cells; ^p < 0.05, ECL- and NAC-exposed cells versus untreated cells; ¤p < 0.05, CSC-exposed cells versus untreated cells; ~p < 0.05, CSC- and UPF1-exposed cells versus untreated cells; "p < 0.05, C- and NAC-exposed cells versus untreated cells. (PDF) [file pone.0142053.s001.pdf]

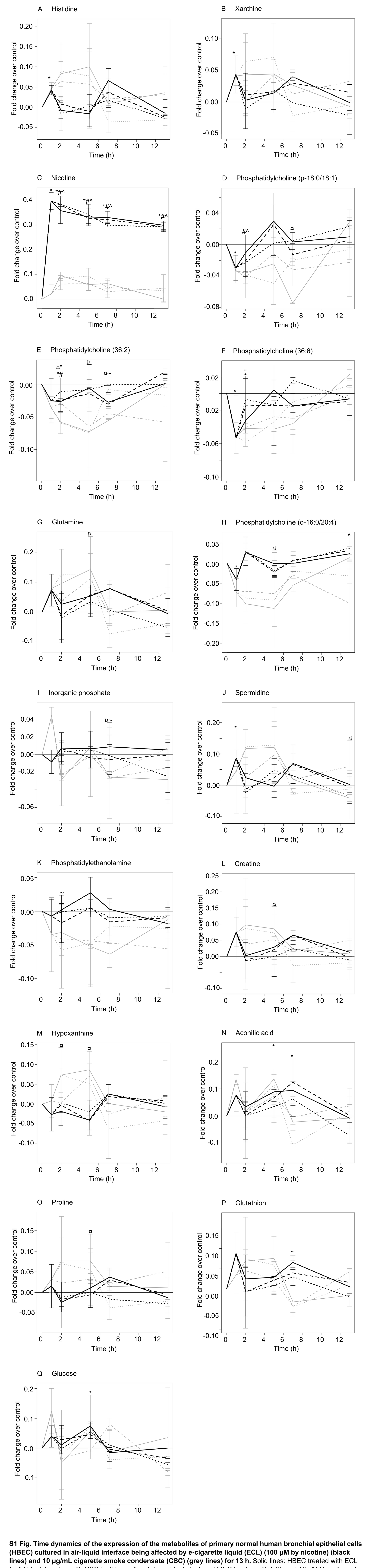


**S1 Fig. Time dynamics of the expression of the metabolites of primary normal human bronchial epithelial cells (HBEC) cultured in air-liquid interface being affected by e-cigarette liquid (ECL) (100 µM by nicotine) (black lines) and 10 µg/mL cigarette smoke condensate (CSC) (grey lines) for 13 h.** Solid lines: HBEC treated with ECL (solid black lines) or with CSC (solid grey lines). Long black dashes: HBEC treated with ECL and 10 µM O-methoxy-L-tyrosinyl-γ-L-glutamyl-L-cysteinylglycine (UPF1) (added at 1h). Long grey dashes: HBEC treated with CSC and 10 µM UPF1 (added at 1h). Short black dashes: HBEC treated with ECL and 2 mM N-acetylcysteine (NAC) (added at 1h). Short grey dashes: HBEC treated with CSC and 2 mM NAC (added at 1h). For each treatment and for any time point, n = 3. Error bars indicate standard errors of means. (A) Histidine ($[M+H]^+$ = 156); (B) xanthine ($[M+H]^+$ = 153); (C) nicotine ($[M+H]^+$ = 163); (D) phosphatidylcholine (p-18:0/18:1) ($[M+H]^+$ = 772); (E) phosphatidylcholine (36:2) ($[M+H]^+$ = 786); (F) phosphatidylcholine (36:6) ($[M+H]^+$ = 778); (G) glutamine ($[M+H]^+$ = 147); (H) phosphatidylcholine (o-16:0/20:4) ($[M+H]^+$ = 768); (I) inorganic phosphate ($[M+H]^-$ = 97); (J) spermidine ($[M+H]^+$ = 146); (K) phosphatidylethanolamine (38:7) ($[M+H]^+$ = 762); (L) creatine ($[M+H]^+$ = 132); (M) hypoxanthine ($[M+H]^+$ = 137); (N) aconitic acid ($[M-H]^-$ = 173); (O) proline ($[M+H]^+$ = 116); (P) glutathione ($[M-H]^-$ = 306; (Q) glucose ($[M-H]^-$ = 179). *p < 0.05, ECL-exposed cells versus untreated cells; #p < 0.05, ECL- and UPF1-exposed cells versus untreated cells; ^p < 0.05, ECL- and NAC-exposed cells versus untreated cells; ¤p < 0.05, CSC-exposed cells versus untreated cells; ~p < 0.05, CSC- and UPF1-exposed cells versus untreated cells; "p < 0.05, C- and NAC-exposed cells versus untreated cells.
